# Silencing of the calcium-dependent protein kinase *TaCDPK27* improves wheat resistance to powdery mildew

**DOI:** 10.1186/s12870-023-04140-y

**Published:** 2023-03-08

**Authors:** Jie-yu Yue, Jin-lan Jiao, Wen-wen Wang, Xin-rui Jie, Hua-zhong Wang

**Affiliations:** Tianjin Key Laboratory of Animal and Plant Resistance, Tianjin Normal University, Tianjin, 300387 People’s Republic of China

**Keywords:** Wheat, Powdery mildew, Programmed cell death, Calcium-dependent protein kinase TaCDPK27, Autophagy

## Abstract

**Background:**

Calcium ions (Ca^2+^), secondary messengers, are crucial for the signal transduction process of the interaction between plants and pathogens. Ca^2+^ signaling also regulates autophagy. As plant calcium signal-decoding proteins, calcium-dependent protein kinases (CDPKs) have been found to be involved in biotic and abiotic stress responses. However, information on their functions in response to powdery mildew attack in wheat crops is limited.

**Result:**

In the present study, the expression levels of *TaCDPK27*, four essential autophagy-related genes (ATGs) (*TaATG5*, *TaATG7*, *TaATG8*, and *TaATG10*), and two major metacaspase genes, namely, *TaMCA1* and *TaMCA9*, were increased by powdery mildew (*Blumeria graminis* f. sp. *tritici*, *Bgt*) infection in wheat seedling leaves. Silencing *TaCDPK27* improves wheat seedling resistance to powdery mildew, with fewer *Bgt* hyphae occurring on *TaCDPK27*-silenced wheat seedling leaves than on normal seedlings. In wheat seedling leaves under powdery mildew infection, silencing *TaCDPK27* induced excess contents of reactive oxygen species (ROS); decreased the activities of superoxide dismutase (SOD), peroxidase (POD) and catalase (CAT); and led to an increase in programmed cell death (PCD). Silencing *TaCDPK27* also inhibited autophagy in wheat seedling leaves, and silencing *TaATG7* also enhanced wheat seedling resistance to powdery mildew infection. TaCDPK27-mCherry and GFP-TaATG8h colocalized in wheat protoplasts. Overexpressed TaCDPK27-mCherry fusions required enhanced autophagy activity in wheat protoplast under carbon starvation.

**Conclusion:**

These results suggested that *TaCDPK27* negatively regulates wheat resistance to PW infection, and functionally links with autophagy in wheat.

## Introduction

Powdery mildew (PM), induced by *Blumeria graminis* f. sp. *tritici* (Bgt) infection, is one of the severe wheat diseases, and it has become a significant threat to wheat production throughout the world [1, 2]. Host resistance is the most efficient and preferred means against PW infection. Plants defense signaling in response to fungal pathogen infection is regulated by at least two primary nodes of the host innate immune system, pathogen-associated molecular pattern (PAMP)‐triggered immunity (PTI) which belongs to non-host (basal) resistance and effector‐triggered immunity (ETI) that can activate robust immune signaling always by inducing hypersensitive response (HR) cell death to prevent pathogen growth and proliferation [3, 4]. The critical factors in the regulation of wheat HR to PM infection are still largely unknown. Therefore, to identify the genes conferring to PW resistance will be necessary and contribute to wheat breeding.

Calcium-dependent protein kinases (CDPKs) are essential components of the calcium (Ca^2+^)-regulated signaling pathway and function in plant responses to abiotic and biotic stresses [5]. CDPKs are directly regulated by Ca^2+^ -controlled protein kinases [6]. Genome studies have shown that plant CDPKs are always a large family. For example, 34 CDPKs, 31 CDPKs, 20 CDPKs, 30 CDPKs, 41 CDPKs and 40 CDPKs have been identified in *Arabidopsis*, rice, bread wheat, poplar, cotton and maize, respectively [7–12]. Growing evidence has confirmed that CDPKs participate in regulating diverse stress responses in plants, including reactive oxygen species (ROS) production included two key members hydrogen peroxide (H_2_O_2_) and superoxide anion (O_2_.^−^), and diverse hypersensitive response (HR) to stress. In wheat, *TaCPK7-D* plays an important role in promoting host resistance to sharp eyespot disease through the modulation of the expression of defense-associated genes [13]. *TaCDPK27* and *TaCDPK34* play positive regulatory role in enhanced salt and drought tolerance of wheat [7, 14]. *TaCDPK2-A* is a key element that confers PW resistance in wheat and promotes bacterial blight resistance in transgenic rice [15]. *TaCDPK13* can increase the expression of the NADPH oxidase gene *TaNOX7* and enhance the production of ROS, and this gene is also involved in wheat growth and development regulation and confers wheat tolerance to drought stress [16]. *TaCDPK5/TaCDPK9-1*, which control the phosphorylation status of TabZIP60, a bZIP transcription factor (TF), are induced by salt, abscisic acid (ABA), and polyethylene glycol (PEG) -induced [6]. *BnaCDPK6L* and *BnaCDPK2* function in regulating ROS and HR-like cell death in rapeseed [17, 18]. Pathogen-triggered HR cell death, a mode of programmed cell death (PCD) in plants, is one of the most efficient and immediate defense responses [19, 20]. Metacaspases (caspase-like proteins, MCAs) are important regulators of stress-induced PCD, and caspase-3-like protease is a crucial executioner of PCD [21]. It has been proven that autophagy has a regulatory function in HR PCD involved in plant innate immunity [22, 23]. ROS induce PCD and autophagy, and are critical for regulating the balance between autophagy and PCD in plant upon various stress treatments. It was found that Ca^2+^ has major effect on the positive and negative regulation of autophagy [24]. *Toxoplasma gondii* TgCDPK3 interacts with the host Atg3 and Atg5, promoting cell-autonomous immunity to restrict the parasitic replication, which is beneficial to establish long-term latency and resist infection of less virulent strains [25]. However, whether a CDPK participates in plant response to PW infection, and whether the regulatory process functionally links with ROS, PCD, and autophagy, have not been clarified.

In plants, autophagy is a highly-conserved mechanism that has an important function in the degradation of cytoplasmic proteins, molecules, and organelles through double-membrane vesicles, referred to as autophagosomes [26]. Autophagy formation involves multiple autophagy-related genes (ATGs) [27]. Under normal conditions, basal autophagy occurs at a low level. To combat various stresses, the level of autophagy is quickly and significantly increased to maintain cellular homeostasis [28]. Accumulating evidence shows that autophagy plays an essential role in plant‒pathogen interactions. For example, *ATG2*- and *ATG18-* mediated autophagy are involved in the negative regulation of powdery mildew resistance and mildew-triggered cell death in *Arabidopsis* [20, 29]. Our previous study also found that *ATG6*-mediated autophagy functions in wheat in response to PW infection; this autophagy has a weak promoting effect in the *Pm21*-induced defense response and negatively functions in the basal resistance of susceptible wheat plants [30]. In tomato, autophagy, as a defense strategy, inhibits leaf curl New Delhi virus (LCNDV) infection by degrading the ToLCNDV protein TrAP [31]. Although there is growing evidence indicating that autophagy performs essential functions in cell death and plant immunity, the underlying mechanism involved remains unclear, and the regulatory mechanism remains largely unclear.

Previously, we identified and cloned the cDNA sequences of TaCDPK27, which positively regulated salt tolerance in wheat seedlings [14]. In this study, we investigated the role of *TaCDPK27* in the plant response to PM infection. We established *TaCDPK27*-silencing wheat seedlings and inoculated with PW causal agent conidia. The result indicated that silencing of *TaCDPK27* confers to wheat resistance to PW infection, and functionally linked with autophagy.

## Results

### *TaATGs* and *TaCDPK27* expression in wheat seedling leaves is induced by PW infection

**Fig. 1:**
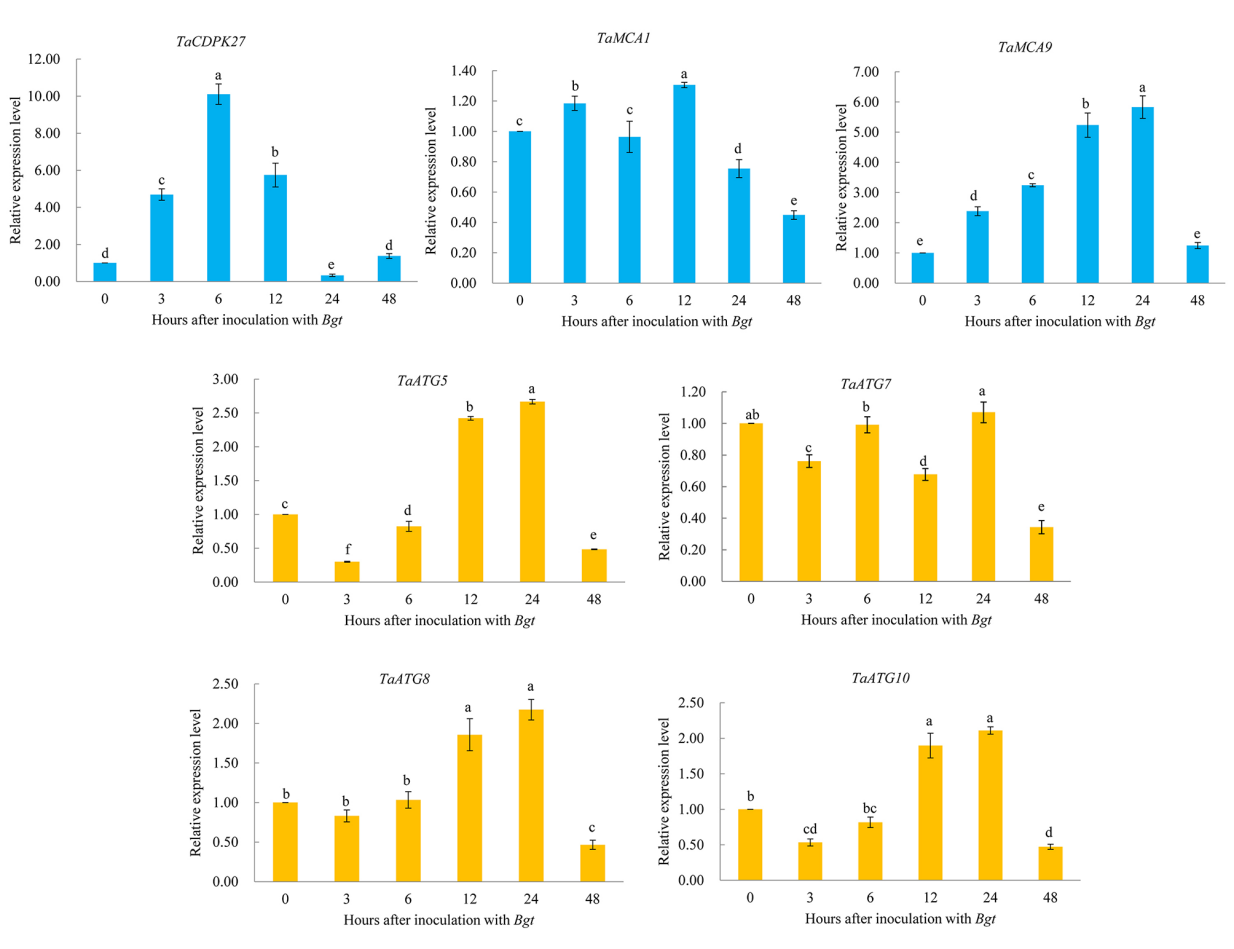
Transcript levels of *TaCDPK27*, key autophagy-related genes (ATGs) and metacaspase (MCA) genes in wheat seedling leaves at 3, 6, 12, 24 and 48 h post-inoculation with powdery mildew causal agent conidia ((*Blumeria graminis* f. sp. *tritici*, *Bgt*)), using qRT-PCR assay. Data are presented as mean ± SD from at least 3 independent experiments. Bars with different letters are significantly different at *P* < 0.05

To investigate whether *TaCDPK27* is involved in the wheat defense response to PW infection, quantitative real-time PCR (qRT-PCR) assay was performed to investigate the expression pattern of *TaCDPK27* after PW infection. The result confirmed that the transcript levels of *TaCDPK27* were significantly elevated in wheat seedling leaves at 3, 6, 12, 24 and 48 h post-inoculation with PW causal agent conidia (Fig. 1). These results suggested that *TaCDPK27* may be involved in wheat anti- powdery mildew responses.

Our previous studies have proven that *TaATG6*-mediated autophagy is involved in the wheat defense response against powdery mildew [30]. Our qRT-PCR results confirmed that other TaATGs, including *TaATG5*, *TaATG7*, *TaATG8*, and *TaATG10* were also significantly upregulated in wheat seedling leaves by PW infection (Fig. 1). Furthermore, PW infection induced the expression of two significant metacaspase genes, *TaMCA1* and *TaMCA9*, involved in regulating PCD in plants (Fig. 1). These results indicated that autophagy and PCD might be involved in regulating the wheat immune response to PW infection.

### Silencing *TaCDPK27* enhances wheat seedling resistance to PW infection

**Fig. 2:**
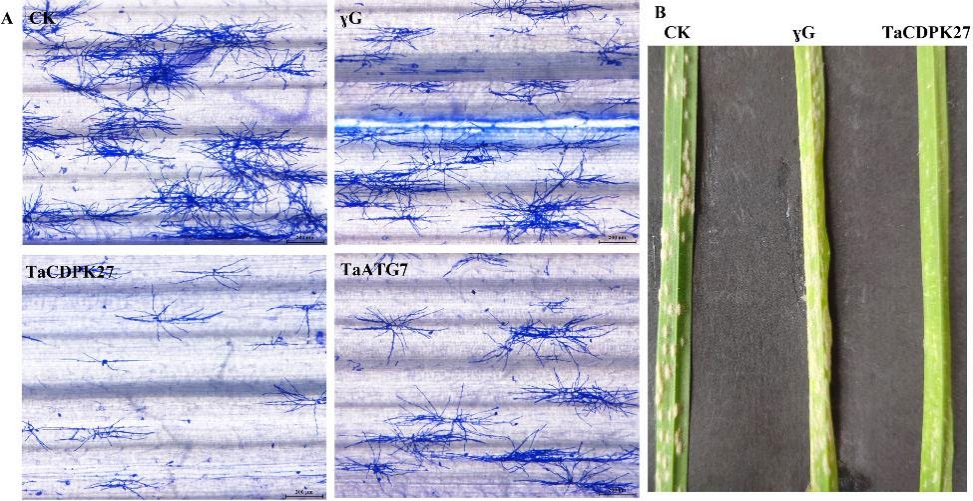
Effect of silencing *TaCDPK27* and *TaATG7*on the wheat seedling resistance response to powdery mildew (*Blumeria graminis* f. sp. *tritici*, *Bgt*) infection. Wild-type (CK) and barley stripe mosaic virus (BSMV)-treated wheat seedlings at the five-leaf stage were inoculated with powdery mildew causal agent conidia. Fungal structures were stained with Coomassie brilliant blue and visualized via microscopy. (**A**) Representative images of *Bgt* infection of the fourth leaves of BSMV-treated wheat seedlings at 3 days after inoculation (Bar = 200 μm). (**B**) Representative images of powdery mildew infection of the fourth leaves of BSMV- inoculated wheat seedlings at 7 days after inoculation

To reveal the possible function of *TaCDPK27* in the wheat immune response to powdery mildew, *TaCDPK27* was silenced via BSMV-VIGS techniques. The third and fourth leaves of wheat seedlings were inoculated with powdery mildew causal agent conidia. The subsequent results indicated that there were fewer *Bgt* hyphae on *TaCDPK27*-silenced and *TaATG7*-silenced wheat seedling leaves than the γG (the wheat seedlings inoculated BSMV-GFP) control (Fig. 2), indicating that the *TaCDPK27*-silenced and *TaATG7*-silenced wheat seedlings had stronger resistance to powdery mildew infection, respectively.

### Silencing of *TaCDPK27* increased ROS accumulation in wheat seedling leaves subjected to PW infection

**Fig. 3:**
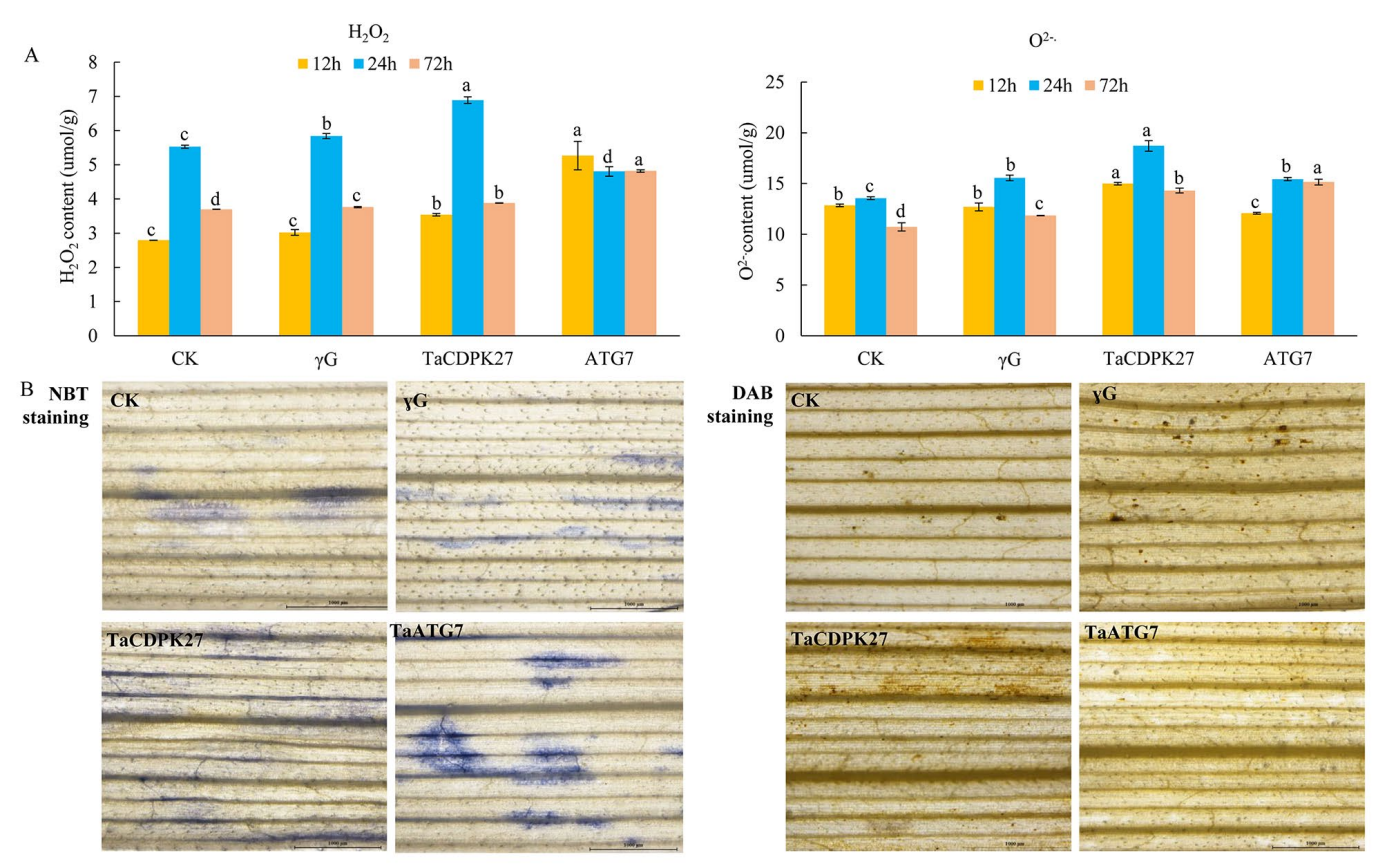
Effect of silencing *TaCDPK27* on H_2_O_2_ and O_2_.^−^ accumulation in wheat seedling leaves infected with powdery mildew (*Blumeria graminis* f. sp. *tritici*, *Bgt*) causal agent conidia. (**A**) Quantitative determination of H_2_O_2_ and O_2_.^−^ accumulation in wheat seedling leaves infected with powdery mildew causal agent conidia. Data are presented as mean ± SD from at least 3 independent experiments. Bars with different letters are significantly different at *P* < 0.05. (**B**) NBT staining showing the generation and accumulation of O^2−^. in leaves of wheat seedlings. DAB staining showing the generation and accumulation of H_2_O_2_ in leaves of wheat seedlings. The CK corresponds to wild-type wheat seedlings, γG corresponds to BSMV-VIGS-GFP-inoculated wheat seedlings, and *TaCDPK27* corresponds to BSMV-VIGS-TaCDPK27-inoculated wheat seedlings. *TaATG7* corresponds to BSMV-VIGS-TaATG7-inoculated wheat seedlings. All the experiments presented here were performed at least three times, and similar results were obtained

**Fig. 4:**
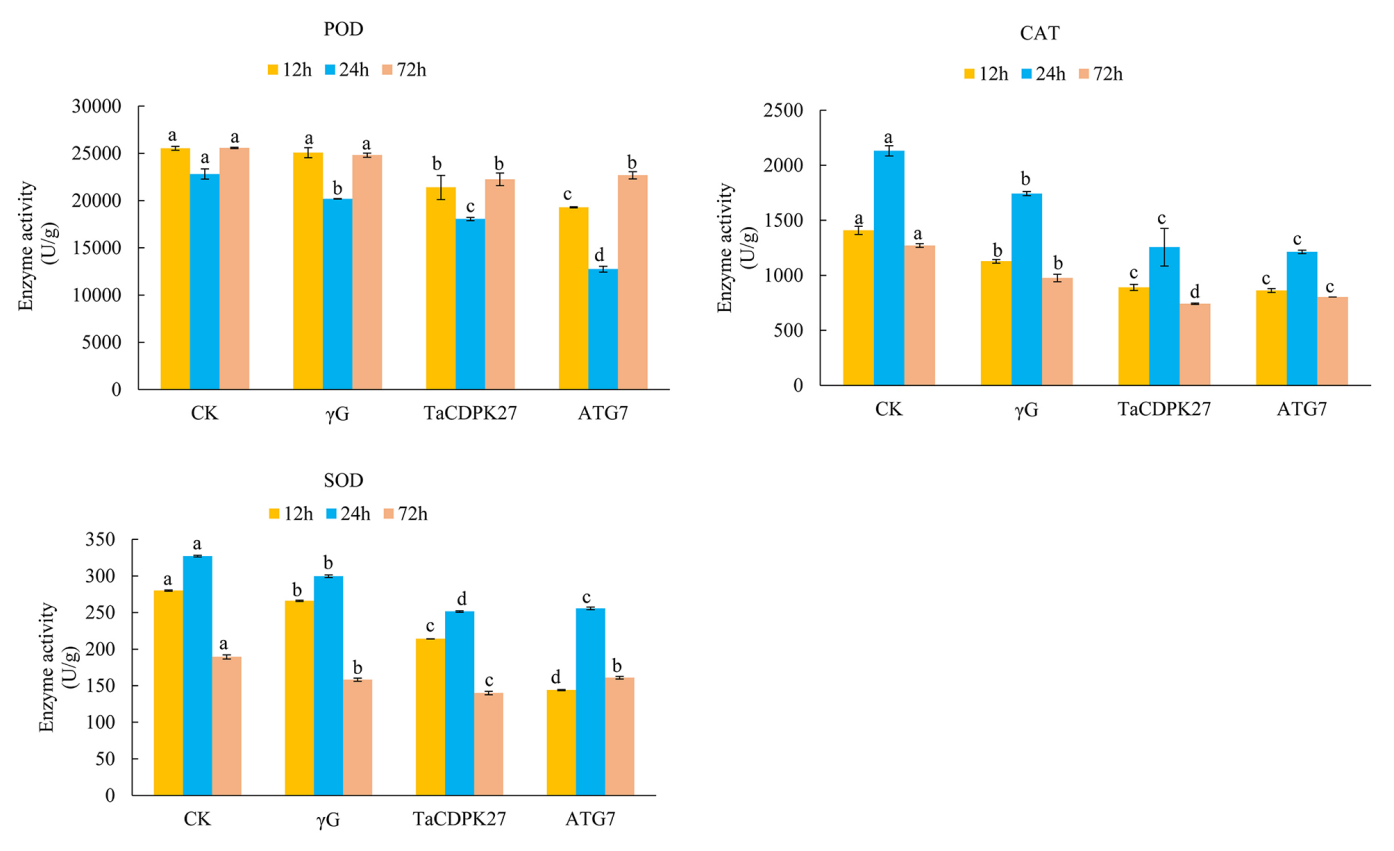
Effect of silencing *TaCDPK27* on the activities of POD, SOD and CAT in wheat seedling leaves infected with powdery mildew (*Blumeria graminis* f. sp. *tritici*, *Bgt*) causal agent conidia. CK corresponds to wild-type wheat seedlings, γG corresponds to BSMV-VIGS-GFP-inoculated wheat seedlings, and *TaCDPK27* corresponds to BSMV-VIGS-TaCDPK27-inoculated wheat seedlings. *TaATG7* corresponds to BSMV-VIGS-TaATG7-inoculated wheat seedlings. The different letters in each treatment show significant differences (*P* < 0.05). All the experiments presented here were performed at least three times, and similar results were obtained

To confirm whether *TaCDPK27* is involved in ROS accumulation in wheat seedling leaves to influence plant resistance, we determined the production of H_2_O_2_ and O_2_.^−^. The results indicated that both H_2_O_2_ and O_2_.^−^ accumulation in the *TaCDPK27*-silenced and *TaATG7*-silenced seedlings was even greater than that in the control seedlings subjected to PW infection (Fig. 3). The staining results were in agreement with the quantitative analysis results of H_2_O_2_ and O^2.−^. Moreover, in agreement with the above results, silencing of *TaCDPK27* and *TaATG7* resulted in a reduction in the activities of POD, SOD and CAT in the wheat seedling leaves subjected to PW infection (Fig. 4). These results suggested that silencing of *TaCDPK27* and *TaATG7* increased excess ROS accumulation via decreasing activities of SOD, POD and CAT under PW infection, and subsequently affected wheat resistance during the PW infection.

### Silencing of *TaCDPK27* enhances PCD in wheat seedling leaves subjected to PW infection

**Fig. 5:**
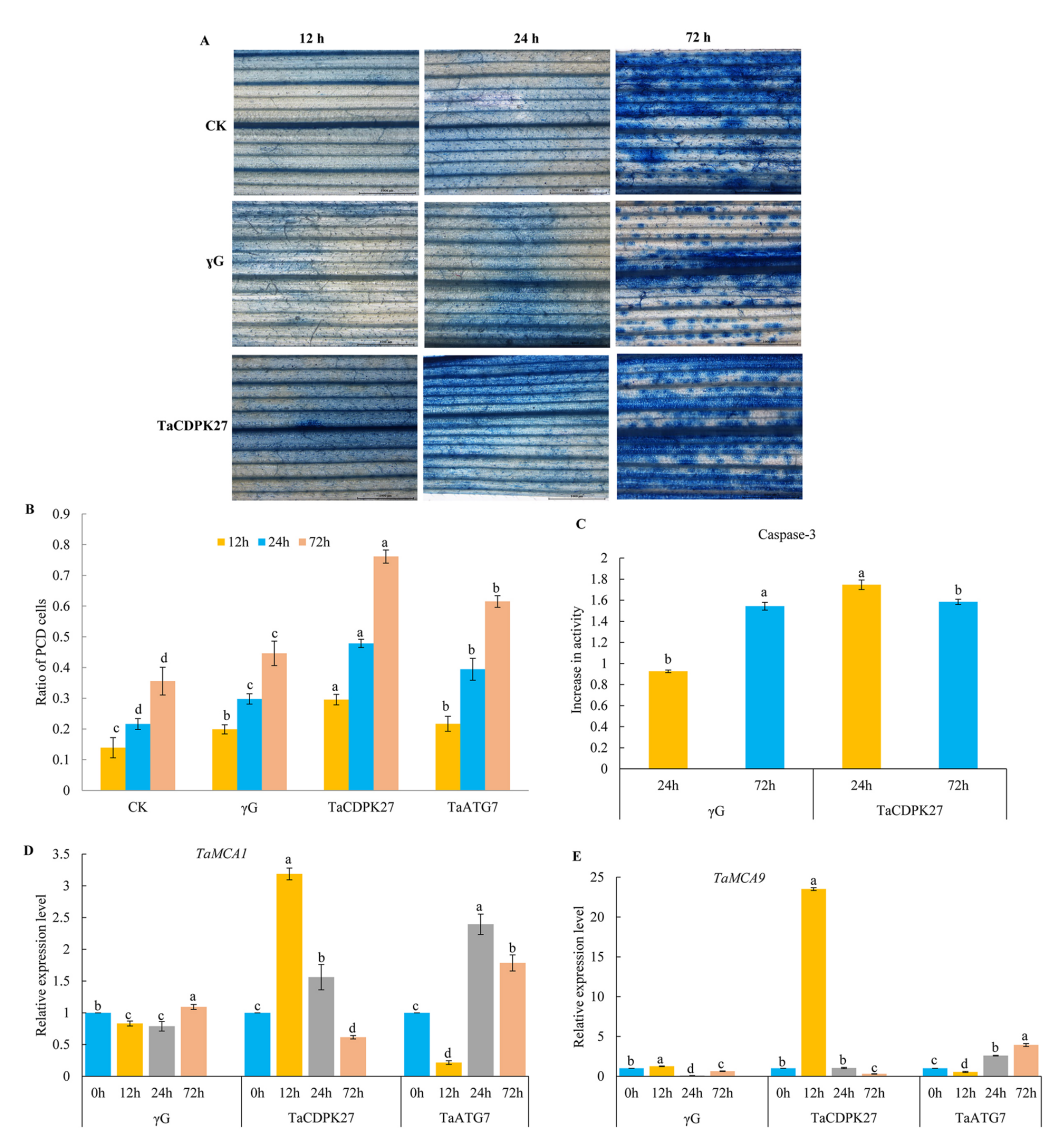
Effect of silencing *TaCDPK27* on cell death in wheat seedling leaves induced by powdery mildew (*Blumeria graminis* f. sp. *tritici*, *Bgt*) infection. (**A**) Evans blue staining showing the cell death. in leaves of wheat seedlings (Bar = 1000 μm). (**B**) The ratio of PCD cells and caspase-3-like activity in leaves of wheat seedlings under powdery mildew infection. (**C**) Transcript levels of *TaMCA1* and *TaMCA9* in wheat seedling leaves under powdery mildew infection. The CK corresponds to wild-type wheat seedlings, γG corresponds to BSMV-VIGS-GFP-inoculated wheat seedlings, and *TaCDPK27* corresponds to BSMV-VIGS-TaCDPK27-inoculated wheat seedlings. *TaATG7* corresponds to BSMV-VIGS-TaATG7-inoculated wheat seedlings. CK corresponds to wild-type wheat seedlings, γG corresponds to BSMV-VIGS-GFP-inoculated wheat seedlings, and TaCDPK27 corresponds to BSMV-VIGS-TaCDPK27-inoculated wheat seedlings. *TaATG7* corresponds to BSMV-VIGS-TaATG7-inoculated wheat seedlings. Data are presented as mean ± SD from at least 3 independent experiments. The different letters in each treatment show significant differences (*P* < 0.05)

To test whether silencing *TaCDPK27* conferred cell death to wheat seedling leaves subjected to PW infection, Evans blue (an indicator of cell death or necrosis) was employed to stain the wheat seedling leaves in situ. Microscope observation indicated that leaf cell death significantly increased in wheat seedlings leaves triggered by PW infection, and silencing *TaCDPK27* aggravated mildew inoculation-triggered cell death in the leaves (Fig. 5A). To determine whether *TaCDPK27* has a regulatory function in PW infection-triggered HR-related PCD, a TUNEL assay was conducted. The results indicated that PW infection promoted PCD in wheat seedling leaves. Silencing of *TaCDPK27* and *TaATG7* further increased the PW infection-induced PCD ratio in the wheat seedlings leaves (Fig. 5B). These results indicated that *TaCDPK27* has a vital regulatory role on the PCD level in wheat seedling leaves subjected to PW infection, which has the same effect of *TaATG7* silencing on the PCD level in wheat seedling leaves subjected to PW infection.

A colorimetric experiment was performed to verify whether *TaCDPK27* has regulatory activity to caspase-3-like activity in wheat seedling leaves after inoculation with PW causal agent conidia for 72 h. Silencing *TaCDPK27* and *TaATG7* triggered an increased level of caspase 3-like activity in wheat seedlings induced by PW infection (Fig. 5C). Silencing *TaCDPK27* and *TaATG7* resulted in upregulation of *TaMCA1* and *TaMCA9* expression induced by PW infection (Fig. 5D and E) in the wheat seedling leaves. In conclusion, these results indicated that *TaCDPK27* negatively regulates HR-related PCD in wheat seedling leaves caused by PW inoculation, which is consistent with the results induced by TaATG7 silencing.

### Silencing of *TaCDPK27* inhibits the autophagy activity in wheat seedling leaves subjected to PW infection

**Fig. 6:**
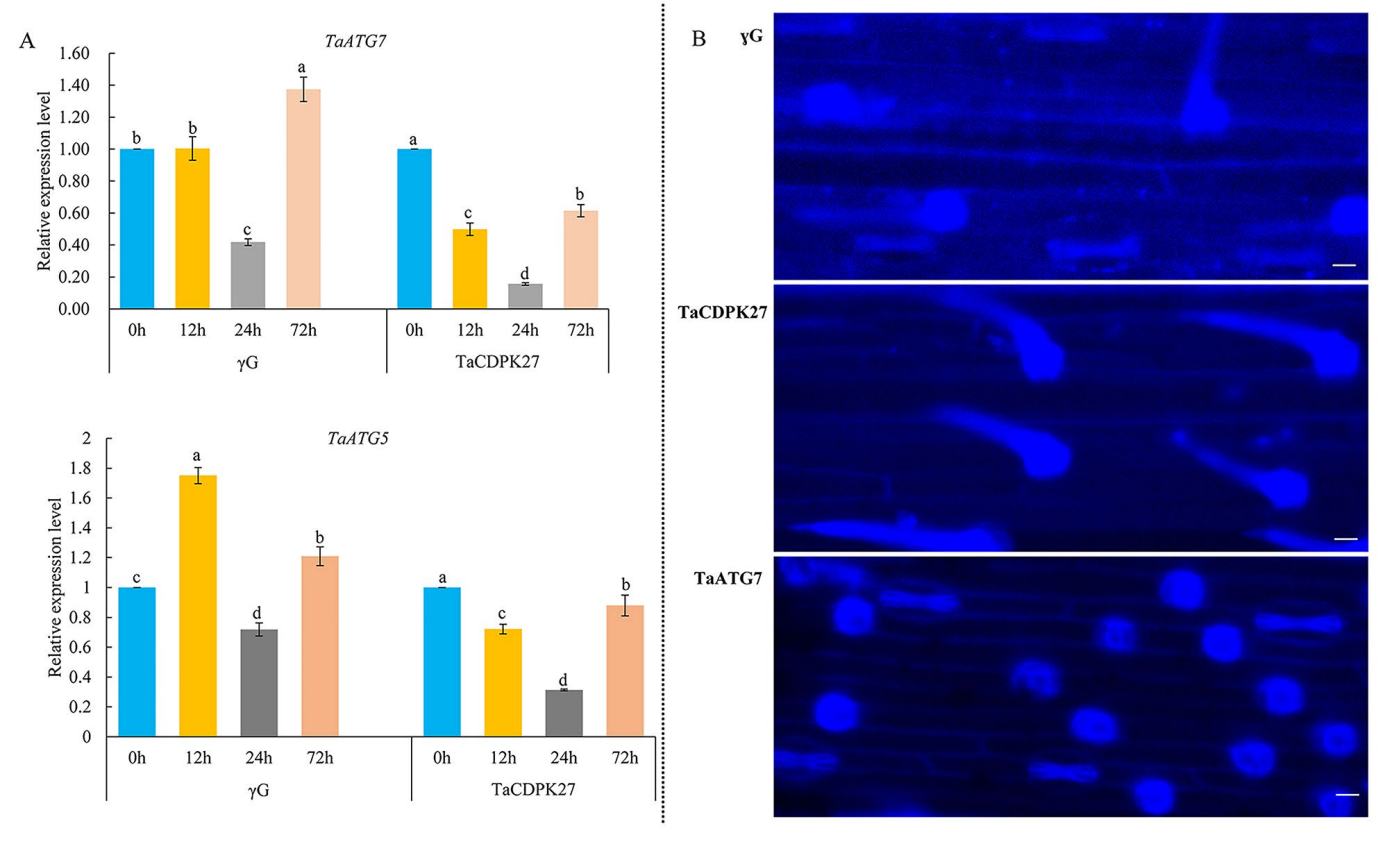
Effect of silencing *TaCDPK27* on autophagy activity in wheat seedling leaves infected with powdery mildew (*Blumeria graminis* f. sp. *tritici*, *Bgt*). (**A**) The effect of silencing *TaCDPK27* on the relative expression analysis of *TaATG5* and *TaATG7* in leaves of wheat seedlings under *Bgt* infection. Data are shown as mean ± SD of three independent experiments. The different letters in each treatment show significant differences (*P* < 0.05). (**B**) The effect of silencing *TaCDPK27* or *TaATG7* on the autophagosomes formation in leaves of wheat seedings under *Bgt* infection which were stained with monodansylcadaverine (MDC). Fluorescence dots are autophagosomes (Bars = 10 μm). γG corresponds to BSMV-VIGS-GFP-inoculated wheat seedlings, and TaCDPK27 corresponds to BSMV-VIGS-TaCDPK27-inoculated wheat seedlings. *TaATG7* corresponds to BSMV-VIGS-TaATG7-inoculated wheat seedlings

After wheat seedlings were incubated with PW for 72 h, silencing *TaCDPK27* suppressed autophagy in the leaves, which was associated with decreased expression of vital ATGs, including *TaATG5* and *TaATG7* (Fig. 6A) and a significant decrease in autophagosomes in wheat seedling leaves in comparison to those in the γG seedlings and WT seedlings (Fig. 6B). To verify whether *TaCDPK27* function in PW infection-induced HR-related PCD is associated with autophagy, we used *TaATG7*-silenced seedlings for PW inoculation. The results showed that silencing *TaATG7* also improves wheat seedling resistance to powdery mildew, with fewer *Bgt* hyphae on *TaATG7*-silenced wheat seedling leaves than on the control seedling leaves (Fig. 2). These results suggested that *TaCDPK27* negatively regulates PW resistance in wheat by suppressing autophagy.

### TaCDPK27 colocalizes with ATG8a under carbon starvation

**Fig. 7:**
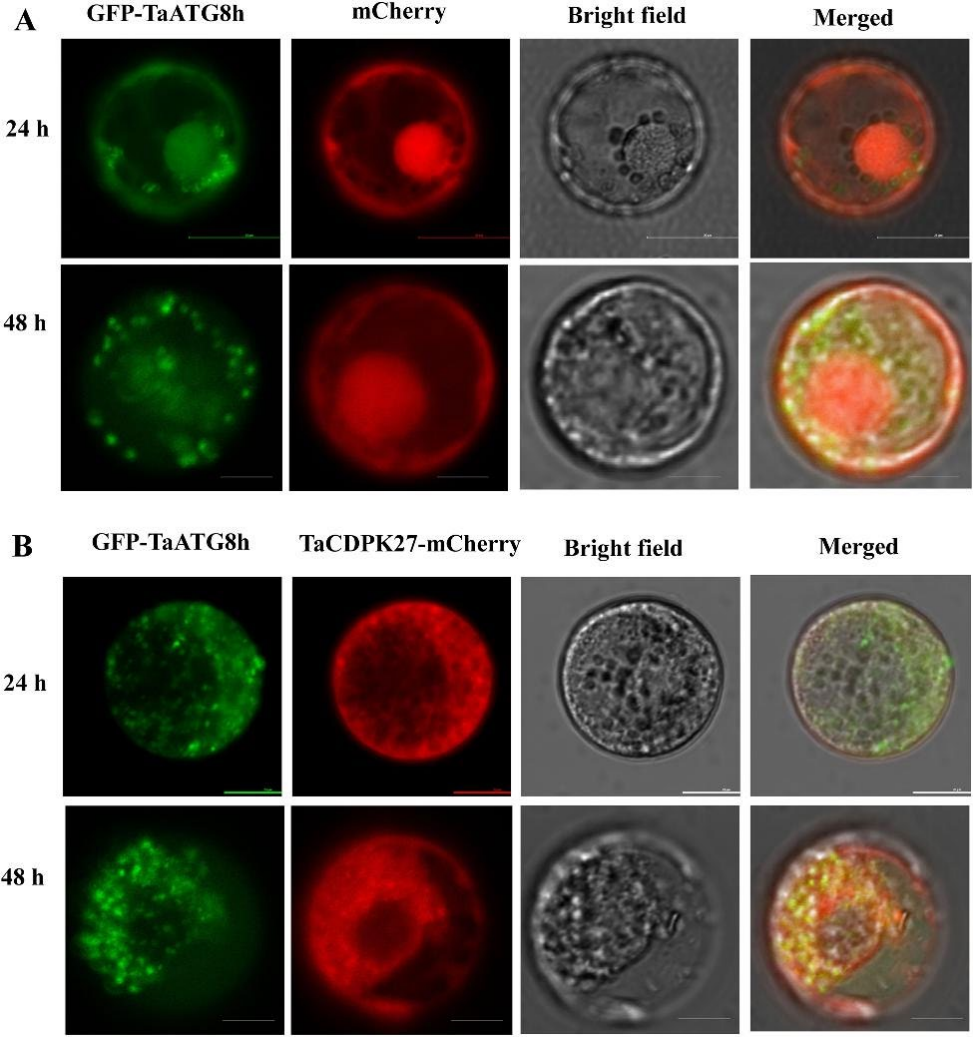
TaCDPK27 colocalizes with ATG8a under carbon starvation. (**A**) TaCDPK27-mCherry colocalizes with GFP-ATG8h in wheat protoplasts (Bar = 25 μm). (**B**) Overexpression of TaCDPK27 enhances autophagy activity in wheat protoplasts (Bar = 20 μm). The protoplasts transfected with mCherry are presented as mCherry. TaCDPK27-mCherry (TaCDPK27-mCherry) and GFP-TaATG8a were coexpressed in these protoplasts to label autophagic structures

We coexpressed TaCDPK27-mCherry and the autophagy marker GFP-TaATG8h in wheat protoplasts. The results showed that TaCDPK27-mCherry-labeled and GFP-TaATG8h-labeled bodies colocalized in the protoplasts, suggesting the degradation of TaCDPK27-mCherry occurs through an autophagy pathway (Fig. 7A). Under carbon starvation, the coexpression of TaCDPK27-mCherry and GFP-TaATG8a triggered an increase in the number of GFP-TaATG8a labeled autophagosomes at 24 and 48 h in each protoplast at compared to the protoplasts that expressed GFP-TaATG8a alone or those that coexpressed mCherry and GFP-TaATG8a. These results suggested that degradation of the overexpressed TaCDPK27-mCherry fusion constructs requires enhanced autophagy activity (Fig. 7B).

## Discission

Wheat is a major crop species worldwide [1]. Powdery mildew is often a destructive disease that results in significant loss of wheat productivity [2]. Understanding the stress response and the adaptive mechanisms in response to powdery mildew infection is essential and fundamental.

CDPKs play important roles in plant immune and stress signaling responses [5, 16, 32–34]. *TaCPK2-A* is required for wheat powdery mildew resistance and overexpressing TaCPK2-A in rice was shown to enhance bacterial blight resistance [15]. *StCDPK5* positively regulates the tolerance of transgenic potato plants containing *PVS3:StCDPK5VK* to late blight pathogens, and has a negative regulatory role in potato resistance to early blight pathogens through an ROS burst [35]. *BnaCPK2* functions in ROS and cell death control by interacting with RbohD in oilseed rape [18]. *CaCDPK15* provides resistance to *Ralstonia solanacearum* infection in pepper plants [36]. We have previously reported the involvement of *TaCDPK27*-mediated calcium signaling in regulating the wheat seedling response to salt stress via PCD [14]. However, the biological functions of *TaCDPK27* in wheat resistance against PW have yet to be elucidated. Based on our results, *TaCDPK27* was shown to be involved in defense signaling and provide PW resistance. In agreement with the above results, silencing *TaCDPK27* led to downregulated expression of *ATGs*, an accumulation of ROS and increased PCD, resulting in increased resistance to PW infection. Combining the results of previous studies and our results, *TaCDPK27* regulates resistance to PW infection through excessive ROS accumulation and its positive role in regulating PCD. Interestingly, the regulatory process of *TaCDPK27* was found to be associated with increased autophagy.

Under biotic stress, HR-related PCD has a vital function in the regulation of plant-pathogen interactions, which are known to limit pathogenic invasion [37]. Previous research has indicated that PCD occurs following autophagy in plants in response to stress conditions [38, 39]. The interactions among autophagy, cell death and disease resistance are still not well understood. Autophagy is an essential catabolic pathway that functions in the degradation and recycling of multiple cytoplasmic components, including proteins, ribosomes and entire organelles, which are sequestered in autophagosomes [40]. Growing evidence has shown that autophagy provides plant tolerance to various stress conditions, such as pathogen attack, drought, salinity and carbon and nitrogen starvation [30, 40]. Autophagy also has pro-survival or pro-death functions in regulating ETI associated HR-related PCD in pathological conditions [30, 41]. Silencing *glyceraldehyde-3-phosphate dehydrogenases* (GAPCs) significantly activated *ATG3*-dependent autophagy, promoted N gene-regulated cell death, and enhanced *Nicotiana benthamiana* plant resistance to incompatible pathogens, including *Tobacco mosaic virus* and *Pseudomonas syringae* pv tomato DC3000, and the compatible pathogen *P. syringae pv tabaci*., supporting the pro-death activity of autophagy [42]. Suppressing *PbrATG5* in pear conferred sensitivity to *Botryosphaeria dothidea* inoculation, also supporting the pro-death activity of autophagy [43]. *ATG2* has a negative role in regulating powdery mildew resistance as well as mildew-induced cell death [20]. Silencing *ATG2* enhanced soybean resistance to *Pseudomonas syringae* pv. *Glycinea* (Psg), with increased accumulation of H_2_O_2_ occurring in soybean leaves [44], which supports the pro-survival activity of autophagy. In this study, silencing *TaCDPK27* aggravated HR-induced PCD, with a reduction in autophagosomes and a decrease in *ATG* expression in wheat seedling leaves subjected to powdery mildew infection. These findings indicated that *TaCDPK27* is involved in regulating HR-related PCD during wheat seedling-PW interactions through inhibition of autophagy, promoting HR-related PCD. ATG proteins are essential regulatory factors in autophagosome formation [27]. Among them, ATG8 family proteins localize to autophagosomal membranes and execute important functions during autophagy in various species [41]. In agreement with this, silencing of *TaCDPK27* or *TaATG7* in wheat seedlings decreased autophagy and increased PCD during powdery mildew infection. Moreover, silencing *TaCDPK27* improved wheat seedling resistance to PW infection by decreasing autophagic activity. These results suggest a link between *TaCDPK27* and autophagy. We further found that TaCDPK27-mCherry colocalized with the membrane associated autophagy marker GFP-TaATG8h in wheat protoplasts, and overexpressed TaCDPK27-mCherry fusions required enhanced autophagy activity in wheat protoplast under carbon starvation.

It is known that one of the necessary responses of plants against pathogen infection is the excess generation of ROS [45]. Plants promote their survival during pathogen infection through enhanced ROS manipulation mechanisms. For example, excessive ROS can induce autophagy and PCD to remove damaged organelles, abnormal proteins and damaged cells that contribute to oxidative stress, restoring ROS to physiological levels and limiting the spread of pathogens [46]. As mentioned above, certain ROS production in wheat leaves is likely a positive defensive role in response to PW infection. However, excessive doses of ROS can cause oxidative damage in the seedling leaves. As a result, autophagy and PCD in leaves could protect wheat seedling leaves from oxidative damage. By silencing *TaATG7* to suppress autophagy in wheat seedling leaves, we found an obvious increase in the number of PCD cells. Silencing *TaCDPK27* induced ROS accumulation, increased PCD, and inhibited autophagy. These results also indicated that, by maintaining the levels of ROS and PCD, autophagy has a positive role in wheat seedling leaves against PW infection, with a decrease in cell oxidative damage and promotion of cell survival. Combining those of previous studies and our results, *TaCDPK27* regulates wheat seedling resistance to powdery mildew through excessive ROS accumulation and positive regulation of PCD. TaCDPK27-mCherry colocalized with GFP-TaATG8h, suggesting that TaCDPK27 may degrade through the autophagy pathway, and thus impeded feedback, which promoted immune responses, including autophagic activity.

**Fig. 8:**
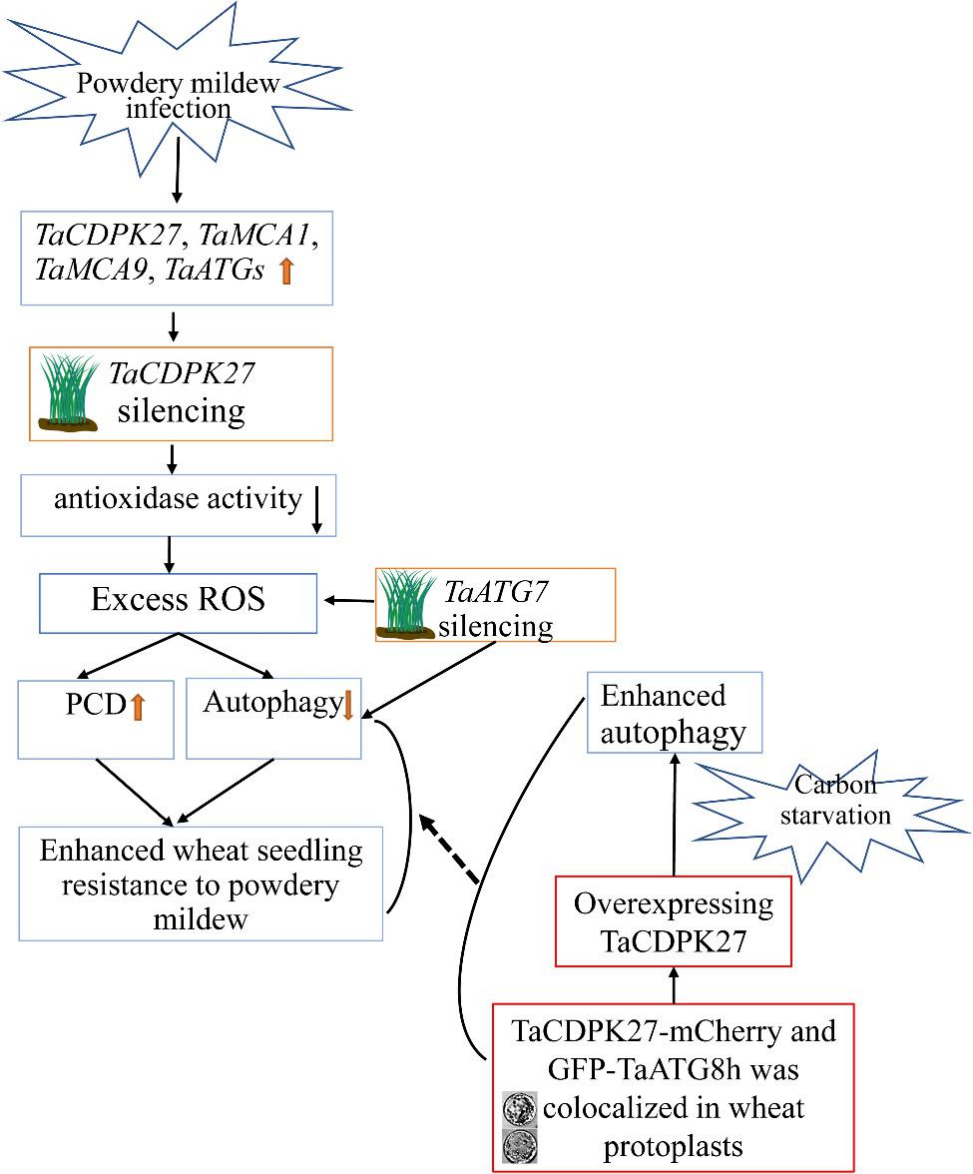
Model of TaCDPK27 function in wheat response to powdery mildew infection

Overall, TaCDPK27 negatively regulates disease resistance to PW infection, by increasing ROS accumulation and PCD via decreased autophagic activity. Silencing autophagy enhanced PCD in wheat seedling leaves infected with PW, supporting the pro-survival activity of autophagy. Moreover, TaCDPK27-mCherry colocalized with the membrane-associated autophagy marker GFP-TaATG8h in wheat protoplasts. The model which connects the *TaCDPK27*, autophagy, ROS accumulation, PCD, and wheat resistance to PW infection was shown as Fig. 8.

## Materials and methods

### Plant variety, growth conditions and powdery mildew inoculation

The wheat cultivar Henong 6425 was chosen as the experimental material and was acquired from Tianjin Academy of Agricultural Sciences. Henong 6425 is susceptible to powdery mildew. The wheat seedlings were grown in hydroponics as described previously [38]. At the two-leaf stage, uniformly growing seedlings were chosen for subsequent PW inoculation. The fresh conidia of *Bgt*, whose virulence type is E09, on heavily diseased seedlings were shaken off onto the second leaves, which were flattened. Leaves were at 0, 3, 6, 12, 24, and 48 h after PW infection, immediately frozen in liquid nitrogen and then stored at -80 °C for subsequent RNA extraction.

A cDNA fragment of 131 bp (+ 1612 bp to + 1742 bp) was employed to acquire the *TaCDPK27*-silenced vector. *TaCDPK27*-silenced wheat seedlings were acquired with barley stripe mosaic virus (BSMV)-based virus-induced gene silencing (VIGS), as described by Yue et al. (2022) [14]. Control (containing wild-type (CK) seedlings and fourth leaf-stage BSMV-VIGS-inoculated seedlings that displayed chlorosis (the materials of which included BSMV-VIGS-GFP-inoculated seedlings (γG) and *TaCDPK27*-silenced seedlings) were inoculated with PW causal agent conidia. Fungal structures were subsequently stained withCoomassie brilliant blue and visualized via microscopy.

### Physiological measurements and histochemical staining

Evans blue staining was employed to determine cell death in wheat seedling leaves [38]. The in situ generation of superoxide radical (O^2−^.) and hydrogen peroxide (H_2_O_2_) in wheat seedling leaves were evaluated by histochemical staining with nitroblue tetrazolium (NBT) and 3, 3’-diaminobenzidine (DAB) staining, respectively [38]. Determination of the the activities of SOD, POD, and CAT and a TUNEL assay were carried out as described by Yue et al. (2021) [38]. The activity of caspase-3 was determined according to Yue et al. (2022) [47].

### RNA isolation and qRT-PCR

The total RNA of wheat seedling leaves was extracted with TRIzol reagent (Invitrogen, USA). First-strand cDNA synthesis, qRT-PCR assays and data analysis were conducted according to the methods of Yue et al. (2018; 2021) [38, 48]. For each experiment, three replicates were included. The primers used are listed in Table 1.

**Table 1:** Primers used in this paper

Term	Gene	Primer sequences (forward/reverse primer)
qRT-PCR	*TaMCA1*	F: GAACGTGACGGCATGGAC
		R: TGGTATGGGAGATCGAGGAC
qRT-PCR	*TaMCA9*	F: CCACCGACTTCAACCTCATCACG
		R: CCGATCTGCTCCTTCTCCTGGTC
qRT-PCR	*TaCDPK27*	F: GCCGCCTTCCAATACTTT
		R: TTATCCTGATCTACTTCGCCTA
Subcellular localization	*TaCDPK27*	F: AAGTCCGGAGCTAGCTCTAGAATGGGCAACGTCTGCGT
		R: GCCCTTGCTCACCATGGATCCGCTATTTGACTTTATGTTCAAG
qRT-PCR	*TaATG5*	F: CCAGAAAGGCCATGGAATCTAAC
		R: GCCTCTTTCAGGGAATTGTTGTA
qRT-PCR	*TaATG7*	F: TGACGTTATCGCTCCTGTTG
		R: ACAGCTGCTCGAGGAATAGC
qRT-PCR	*TaATG10*	F: TATTACTCGAGAGGAGCATCCCCAC
		R: GATTTTCAATCCTACTGCCTGACCG
qRT-PCR	*TaATG8*	F: GGAAAGGAGGCAAGCTGAA
		R: GCATCTCGTTAGGGACAAGGTA
BMSV-VIGS	TaCDPK27	F: CAAACATTTTTTTTTTTTTTTAGCTAGCGCCGCCTTCCAATACTTT
		R: GATTCTTCTTCCGTTGCTAGCTTATCCTGATCTACTTCGCCTA
BMSV-VIGS	forward fragment of *ATG7*	F: CGCGGATCCGGGAAGTTGTCTAGTGCTCG
		R: GGACTAGTCCCAAGAGTTCCAGCACCTA
BMSV-VIGS	reverse fragment of *ATG7*	F: ACGCGTCGACGGGAAGTTGTCTAGTGCTCG
		R: GGACTAGTCCCAAGAGTTCCAGCACCTA

### Plasmid constructs

GFP-TaATG8h-expressing vectors were constructed according to the methods of Pei et al. (2014) [49]. A pAN583:TaCDPK27-mCherry-expressing vector was constructed as described by Ni et al. (2022) [50]. The primers used are listed in Table 1. The PEG-mediated transfection method was employed to transform the TaCDPK27-mCherry vector and GFP-TaATG8h-expressing vectors into wheat protoplasts [50]. Then, the mCherry and GFP signals were observed using a confocal laser scanning microscope (Nikon Ti2 Eclipse A1).

### Data analysis

Each result is presented as the mean ± standard deviation (SDof three independent experiments. The error bars in the graphs showthe mean of the standard errors. The data analyses were carried out with SPSS software via Duncan’s multiple-range test. *P* values < 0.05 represented the statistical significance threshold.

## Data Availability

The data that support the findings of this study are included in this article.
